# The enigmatic seminal plasma: a proteomics insight from ejaculation to fertilization

**DOI:** 10.1186/s12958-018-0358-6

**Published:** 2018-04-28

**Authors:** Luna Samanta, Rajeshwari Parida, Tania R. Dias, Ashok Agarwal

**Affiliations:** 1 American Center for Reproductive Medicine, Cleveland Clinic, 10681 Carnegie Avenue, Desk X11, Cleveland, OH 44195 USA; 2 Redox Biology Laboratory, Department of Zoology, School of Life Sciences, Ravenshaw University, Cuttack, Odisha 753003 India; 3 Universidade da Beira Interior, 6201-001 Covilhã, Portugal; 4 Department of Microscopy, Laboratory of Cell Biology, Institute of Biomedical Sciences Abel Salazar and Unit for Multidisciplinary Research in Biomedicine, University of Porto, 4050-313 Porto, Portugal; 5 LAQV/REQUIMTE - Laboratory of Bromatology and Hydrology, Faculty of Pharmacy, University of Porto, 4050-313 Porto, Portugal

**Keywords:** Seminal plasma, Exosomes, Prostasomes, Epididymosomes, Cytokines, Female reproductive tract, Male infertility, Proteomics, Assisted reproductive technologies

## Abstract

**Background:**

The ‘omics’ approach for a noninvasive diagnosis of male reproductive system disorders has gained momentum during the last decade, particularly from a screening and prognosis point of view. Due to the rapid development in assisted reproductive technologies (ART) over the years, the major focus of proteomic studies has been around the ejaculated spermatozoa. Although seminal plasma is not a requirement for ART, the question arose whether the role of seminal plasma is merely to transport spermatozoa.

**Main body:**

Seminal plasma (SP) contains a large diversity of proteins that are essential not only for sperm transport, but also for sperm protection and maturation. Most of the proteins bind to sperm surface through exosomes (epididymosomes and prostasomes), modulating sperm function, interaction with the female reproductive tract and finally fertilization. This review focuses on the state-of-art discoveries regarding SP proteome and its role in fertilization.

**Conclusion:**

Tissue-specific proteins in the SP have emerged as fundamental contributors for protein biomarker discovery. This is important for a noninvasive diagnosis of male infertility and development of new therapeutic approaches. Moreover, ART success rates may be improved by taking into account the critical role of seminal proteome in fertilization.

## Background

Semen comprises about 5% of testicular secretions containing spermatozoa and 95% of seminal plasma (SP), which includes secretions from accessory sex glands. The use of assisted reproductive technologies (ART) facilitated the direct involvement of sperm in fertilization, specifically for the delivery of paternal genetic content and embryo development. Consequently, the role of SP in the regulation of reproductive processes to achieve a successful birth has been largely neglected. Although SP components have a potential diagnostic value, such as measurement of fructose levels for abnormalities in seminal vesicles [1] and measurement of prostatic acid phosphatase for the presence of prostatic secretions in SP [2], they have been given little clinical consideration. SP plays a key role in natural fertilization as it contributes to a timely capacitation, acrosome reaction and sperm-oocyte interaction [3, 4]. Its complex proteomic composition, growth factors and transcription factors, nourishes and protects spermatozoa as they travel throughout the male and female reproductive tracts. As a result, it ensures the interaction of spermatozoa with the oocyte to undergo fertilization [5]. The presence of tissue-specific proteins in the SP can serve as potential biomarkers for the assessment of male fertility. One comprehensive review by Drabovich et al. [6] reported the role of SP proteins as markers of male fertility evaluation with special reference to prostate cancer and azoospermia. The various milestones in our understanding of SP proteome (Table 1) have prompted many investigators to gain further insights into its function and role in human reproduction, as well as its potential as a screening, diagnostic and prognostic tool in male fertility assessment. Therefore, this review focuses on the role of SP proteins in different reproductive processes including the modulation of spermatozoa functions and fertilization.

**Table 1:** Major breakthroughs in the development of proteomic biomarkers through proteomic analysis of human seminal plasma

Year	Technique used	Outcome	Reference
1888	Acetic acid precipitation	Detection of propeptone	[16]
1942	Electrophoresis (Tiselius apparatus)	First electrophoretic separation that identified 4 protein fractions	[18]
1942	Electrophoresis (Tiselius apparatus)	Electrophoretically separated 4 protein fractions correspond to albumin, α-, β- and γ-globulins	[17]
1978	2DE	Separated 40 peptides and identified p30 as a marker for semen in forensic samples (vaginal swabs). Established the idea that semen proteins can act as biomarkers	[19]
1981	2DE	Detected > 200 peptides and reported the absence of many glycoproteins in vasectomized men	[20]
2003	2DE-MALDI-TOF-MS	Detected impaired spermatogenesis-associated markers by narrow immobilized pH gradients in azoospermic men	[21]
2007	2DE-LC-MS/MS	Reported candidate marker proteins for non-obstructive and obstructive azoospermia	[26]
2009	1DE-LC-MS/MS	Proposed that downregulation of DJ-1 is responsible for oxidative stress and thereby affects the quality of the semen in asthenozoospermia	[27]
2011	N-linked glycosylated peptide enrichment, combined with LC-MS/MS	N-glycosylated prostate-specific antigen is known to be an efficient biomarker that can distinguish benign prostate hyperplasia from prostate cancer	[30]
2012	2DE-LC-MS/MS	Identified 59 proteins in seminal plasma as candidate biomarkers of prostatitis	[28]
2013	LC-MS/MS	Testis-specific TKTL1, LDHC and PGK2 could distinguish semen from fertile and infertile men	[23]
2013	1DE-LC-MS/MS	Identified proteins that are over- or underexpressed in the seminal plasma of teratozoospermic, oligozoospermic and oligoteratozoospermic men	[29]
2014	LC-MS/MS followed by MS-based multiplex SRM assay	ECM1 and TEX101 levels can distinguish OA from NOA and circumvent testicular biopsies for prediction of outcome of sperm retrieval in azoospermic patients	[6]
2015	1DE-LC-MS/MS	MME and FAM3D along with ROS levels in the seminal plasma may serve as good markers for diagnosis of male infertility	[24]
2016	LC-MS/MS	Cab45/SDF4, protein lefty-1, DNase I, PAP2-alpha, IBP-7, HDC, and CRISP-3 are proposed as putative biomarkers in adolescents with varicocele	[25]

*Abbreviations*: *1DE* one-dimensional electrophoresis, *2DE* two-dimensional electrophoresis, *Cab45/SDF4* 45 kDa calcium-binding protein, *CRISP-3* Cysteine- rich secretory protein 3, *ECM1* extracellular matrix protein 1, *HDC* Histidine decarboxylase, *FAM3D* family with sequence similarity 3 member D, *IBP-7* Insulin-like growth factor-binding protein 7, *LC* liquid chromatography, *LDHC* lactate dehydrogenase C, *MALDI* matrix-assisted laser desorption/ionization, *MME* membrane metalloendopeptidase, *MS* mass spectrometry, *MS/MS* tandem mass spectrometry, *NOA* non-obstructive azoospermia, *OA* obstructive azoospermia, *PAP2* Prostatic acid phosphatase type 2, *PGK2* Phosphoglycerate kinase 2, *ROS* reactive oxygen species, *SRM* selected reaction monitoring, *TEX101* Testis-expressed protein 101, *TKTL1* Transketolase-like protein 1, *TOF* time-of-flight

## Review criteria

PUBMED and Google Scholar databases were explored for relevant literature (1942–2017) on the proteomics study of SP using the following keywords: “seminal fluid proteins”, “semen proteins”, “seminal plasma proteins”, “proteomics and biomarker discovery”, “seminal plasma proteomics”, “seminal plasma biomarkers”, “seminal plasma and sperm”, “seminal plasma and male fertility”, “seminal plasma and inflammation”, “seminal plasma and immune tolerance”, “seminal plasma and ovarian function”, “seminal plasma and female reproductive tract events”, “seminal plasma and endometrial tissue remodeling”, “seminal plasma and uterine receptivity” and “seminal plasma and embryotrophic cytokines”. All full-text articles published in peer-reviewed journals in English were selected, total of 262 articles. Many review articles and animal studies were not considered either due to poor relevance to our topic or repeated information in more recent studies. A total of 99 references were included (97 articles and two book chapters). Nine references with more than 20 years were considered either due to historical importance or as supportive evidence. The majority of the references (61) are from the last 10 years.

### Composition of seminal plasma

**Fig. 1:**
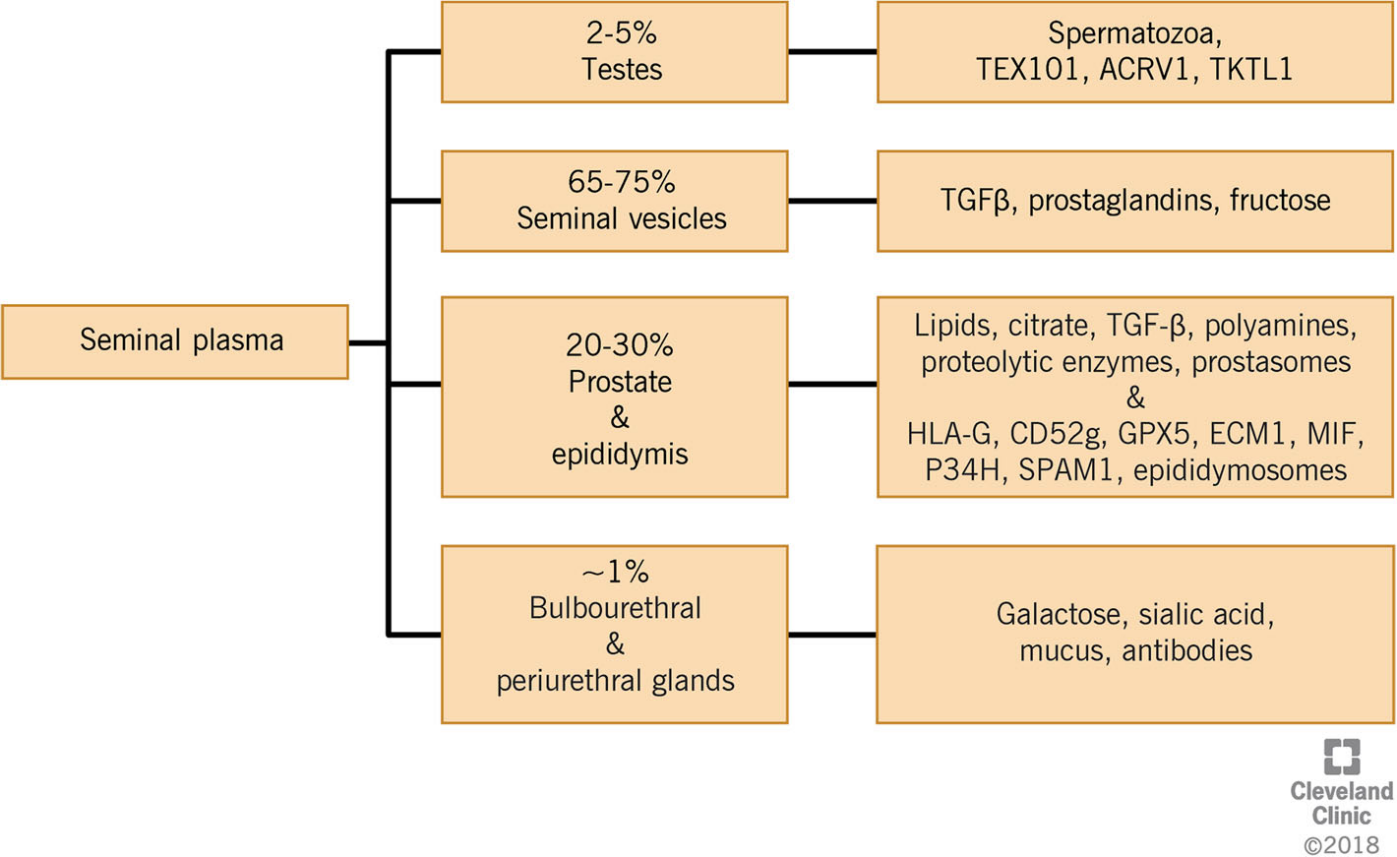
Composition of seminal plasma including the secretions from testes, epididymis, seminal vesicles, prostate, and bulbourethral and periurethral glands

SP is constituted by secretions derived from testes (~ 2–5%; sperm cells), epididymides and prostate (~ 20–30%), seminal vesicles (~ 65–75%), as well as bulbouretheral and periurethral gland (~ 1%) (Fig. 1) [7]. It is rich in sugars, oligosaccharides, glycans [8], lipids [9], inorganic ions, small molecule metabolites [10], cell-free DNA [11], RNA, microRNAs [12], peptides and proteins [10]. The average protein concentration in SP is about 35–55 g/L. These components mediate the interaction between SP and spermatozoa, regulating their function and facilitating their transit through the female reproductive tract [13, 14]. The alkaline secretions of seminal vesicles and prostate counteract the vaginal acidity for optimal sperm survival. The overall contribution of seminal vesicles to SP is the highest in terms of molecular content and includes cytokines, prostaglandins and fructose [15], while the prostate gland secretions are rich in lipids, citrate and proteolytic enzymes [2]. Basic polyamines, namely, spermine, spermidine and putrescine maintain the alkalinity of the semen. Galactose, sialic acid and mucus secreted by the bulbourethral glands act as lubricants for efficient sperm transfer (Fig. 1) [7].

### History and evolution of the study of seminal plasma proteome

In 1888, a report by C. Posner regarding the presence of proteose or propeptone (cocktail of proteolytic digested products) in SP, marked the beginning of seminal plasma proteomics (Table 1) [16, 17]. However, the first electrophoretic separation of SP proteins was in 1942 using a Tiselius apparatus, where two water-soluble fractions including one non-heat-coagulable proteose and a glycoprotein, and two water-insoluble fractions were identified. They also confirmed the content of albumin in seminal plasma to be less than 0.02% and that of nucleoprotein to be less than 0.04% [18]. In the same year, Gray and Huggins [17] reported that the 4 fractions yielded by electrophoretic separation of SP correspond to albumin, α-globulin, β-globulin, and γ-globulin of normal serum. Sensabaugh [19] was able to separate 40 peptides by SDS-PAGE, purify and characterize the semen specific protein p30, which could be used as an alternative test for the forensic identification of semen based on the detection of this protein. Thus, the foundation for the use of seminal proteins as biomarkers was established. Over the years, there has been a tremendous advance in the analytical tools used to decipher the proteome profile of human samples. These advances also enabled the identification of a large number of proteins at a much faster pace (Table 1). More than 200 peptides were detected in the SP of normal fertile men using 2D-electrophoresis, while their vasectomized counterparts lacked a series of glycoproteins. Besides, immunodetection revealed the presence of a number of serum proteins as well as prostatic acid prosphatase and creatine kinase [20]. Subsequently, Starita-Geribaldi et al. [21] developed a narrow immobilized pH gradient covering one pH unit for human SP proteomic analysis, which was followed by matrix-assisted laser desorption ionization (MALDI) and tandem mass spectrometry (MS-MS). These techniques increased the accuracy of differential expression analysis and identification of markers associated with impaired spermatogenesis. Separation of SP proteins from a pool of five samples by gel electrophoresis (1D and 2D) and identification either by matrix-assisted laser desorption ionization-time-of-flight-mass spectrometry (MALDI-TOF-MS) or capillary liquid chromatography tandem mass spectrometry (LC-MS/MS) revealed 100 peptides and proteins [22]. A total of 923 SP proteins including proteases of extracellular origin and other proteins originating from male accessory glands were identified by LC-MS/MS from a single sample [14]. Rolland et al. [23] identified 699 proteins in SP and predicted the presence of a total of 2545 distinctive proteins by comparing their results with previous studies. From their data, the authors also confirmed the presence of proteins from testes (83), epididymis (42), seminal vesicles (7) and prostate (17). Beginning with a moving boundary electrophoresis using a Tiselius apparatus, proteomic research has undergone remarkable evolution over time with the development of high throughput technologies such as LC-MS/MS and with the inclusion of targeted proteomics and possibility to detect post-translational modifications (PTMs) in proteins to assess their functionality. Studies by various groups have recorded thousands of proteins in SP and these were consolidated by Drabovich et al. [6], amounting to 3200 in total. These studies and the advances in proteomic technologies contributed to the recent discovery of key proteins associated with male infertility [24, 25], which may be used as biomarkers for clinical application.

### Proteomic signatures of seminal plasma in evaluation of male infertility

The past decade witnessed an enormous rise in the number of reports on comparative proteomics of SP to identify putative protein markers for various reproductive conditions such as infertility in general [23], obstructive and non-obstructive azoospermia [6, 26], asthenozoospermia [27], prostatitis [28], oxidative-induced infertility [24, 29], varicocele [25], and prostate cancer and benign prostate hyperplasia [30] (Table 2).

**Table 2:** Potential protein markers in seminal plasma under altered pathophysiology

Condition	Identified protein markers	Reference
Poor semen parameters/azoospermia	PGDS	[31, 32]
Obstructive and non-obstructive azoospermia	TEX101, ECM1	[33]
Azoospermia vs normo-, astheno- and oligozoospermia	Fibronectin, PAP, PIP, B2M, PSMA3, LGALS3BP, CNDP2	[13]
Infertility	Semenogelin I/II, olfactory receptor 5R1, Lactoferin, hCAP18, Spindling 1, Clusterin	[3]
Abnormal semen parameters, ROS and asthenozoospermia	DJ-1	[27, 29]
ROS and prostate cancer	LGALS3BP	[37]
Different levels of ROS and infertility	MME, FAM3D	[24]
Fertile vs infertile	TKTL1, LDHC, PGK2	[23]

Seminal plasma proteins as potential biomarkers in several infertile conditions. These biomarkers can be targeted for seminal analysis in clinical research for male fertility evaluation

*Abbreviations*: *B2M* beta-2-microglobulin, *CNDP2* cytosolic nonspecific dipeptidase, *ECM1* extracellular matrix protein 1, *FAM3D* family with sequence similarity 3 member D, *hCAP18* human cationic antimicrobial protein, *LDHC* lactate dehydrogenase C, *LGALS3BP* galectin 3 binding protein, *MME* membrane metalloendopeptidase, *PAP* prostatic acid phosphatase, *PGK2* phosphoglycerate kinase 2, *PIP* prolactin-inducible protein, *PSMA3* proteasome subunit alpha type-3, *PGDS* prostaglandin-D synthase, *ROS* reactive oxygen species, *TEX101* testis-expressed protein 101, *TKTL1* transketolase-like protein 1

A member of the lipocalin superfamily, prostaglandin-D synthase (PGDS) is an extracellular transport protein that includes retinol-binding protein and shows high binding affinity for specific cell receptors and small hydrophobic ligands. PGDS was reported to be associated with poor semen parameters such as decreased sperm count, percentage of motility, and percentage of normal morphology. The expression of this protein showed a gradual decrease from normal to oligospermic, azoospermic and patients who underwent vasectomy [31]. Upon further investigation, PGDS was proposed as a biomarker of obstructive azoospermia [32]. The same group developed a multiplex selected reaction monitoring (SRM) assay to detect the level of testis-expressed protein 101 (TEX101) and epididymis-expressed extracellular matrix protein 1 (ECM1) to distinguish men with obstructive azoospermia from those with non-obstructive azoospermia (Table 2). This technique circumvented the clinical evaluation using the invasive technique of testicular biopsy [33].

A comparative proteomic study by Davalieva et al. [13] using two-dimensional differential gel electrophoresis (2D-DIGE) showed overexpression of 8 proteins: fibronectin, prostatic acid phosphatase (PAP), prolactin-inducible protein (PIP), beta-2-microglobulin (B2M), proteasome subunit alpha type-3 (PSMA3), cytosolic nonspecific dipeptidase (CNDP2), and galectin-3-binding protein (LGALS3BP) in the SP of azoospermic men when compared with at least one of the other studied groups (normozoospermic, asthenozoospermic and oligozoospermic). In particular, PAP was significantly overexpressed in azoospermic patients relative to all the other groups. Milardi et al. [3] evaluated the seminal proteomic profile among a pool of five men with proven fertility and proposed a panel of proteins of importance for male fertility such as semenogelin I, semenogelin II, olfactory receptor 5R1, lactoferrin, human cationic antimicrobial protein (hCAP18), spindling 1, and clusterin. Semenogelins are the main proteins responsible for the formation of a gel matrix that entraps spermatozoa into a seminal coagulum right after ejaculation. This has a particular importance for sperm function because semenogelins act as natural regulators of sperm capacitation as they prevent its premature occurrence [34]. High concentrations of semenogelins were also suggested as a biomarker in asthenozoospermic men [35]. The hCAP18 is also expressed by the epididymal epithelium and in seminal plasma at high concentrations and has a key role in the innate immunity of the male reproductive system [36].

Stress proteins such as DJ-1 were found to be differentially regulated and expressed in men with: (1) normal sperm count and abnormal morphology, (2) oligozoospermia and normal morphology, and (3) oligozoospermia and abnormal morphology, when compared to normozoospermic samples. These proteins may serve as a potential biomarkers to identify the underlying mechanisms that lead to poor sperm quality in these men [37]. Wang et al. [27] proposed that the downregulation of DJ-1 induces oxidative stress, thereby affecting the quality of semen in asthenozoospermic men. A comparative proteomics study using semen samples with normal or high levels of reactive oxygen species (ROS) indicated that the LGALS3BP, which is responsible for host defense against viruses and tumor cells, was exclusively detected in the group with normal ROS levels. Thus, LGALS3BP could be a candidate marker for the diagnosis and treatment of patients with cancer of the reproductive tract [29]. Further in-depth investigation involving SP from fertile and infertile men with varying levels of ROS (low, medium and high) showed that the expression profile of proteins involved in PTMs, protein folding and transport were severely altered in the infertile group. Furthermore, two proteins were identified as putative markers: the membrane metalloendopeptidase (MME) and family with sequence similarity 3 member D (FAM3D).The former was consistently overexpressed in all three groups of infertile men, whereas the latter was uniquely expressed in the fertile group [24]. A combined sample pre-fractionation using peptide ligand library columns followed by shotgun nano-LC-MS/MS analysis of the tryptic peptides corresponding to each elution fraction, enabled the identification of 669 proteins. Among those, three important proteins: transketolase-like protein 1 (TKTL1), L-lactate dehydrogense C chain (LDHC) and phosphoglycerate kinase 2 (PGK2), were also proposed as biomarkers of fertility [23].

SP has also a high expression of cytokines that are associated with male infertility [38]. In the male genital tract, there is a tissue-specific secretion of cytokines and chemokines into the SP. The epididymis mainly secretes soluble HLA-G and CD52g, whereas the seminal vesicles are the principal source of prostaglandins and transforming growth factor β (TGF-β). The prostate also produces TGF-β and other bioactive components including small RNAs. The penile urethra is primarily responsible for the production of local antibodies in the male genital tract [5, 39, 40].

The studies presented in our review provide an evidence that SP proteome is vast and may have a role beyond spermatozoa transport. Although these mechanisms are not completely understood, the identified proteins may serve as biomarkers for the development of new diagnostic and therapeutic tools. For instance, a testis-secreted acrosomal vesicle protein 1 (ACRV1, SP-10) associated with sperm-zona binding and penetration [41] has been the basis for the development of an immunodiagnostic home test, SpermCheck Fertility® (Bio-Rad Laboratories, Hercules, CA), to detect the sperm concentration in semen. In this qualitative test, the number of sperm is directly proportional to the signal strength of ELISA assays for the measurement of SP-10 [42]. Similar approaches can be used to develop new technologies for the clinical evaluation of male infertility.

### Seminal exosomal proteins and their role in sperm function

**Fig. 2:**
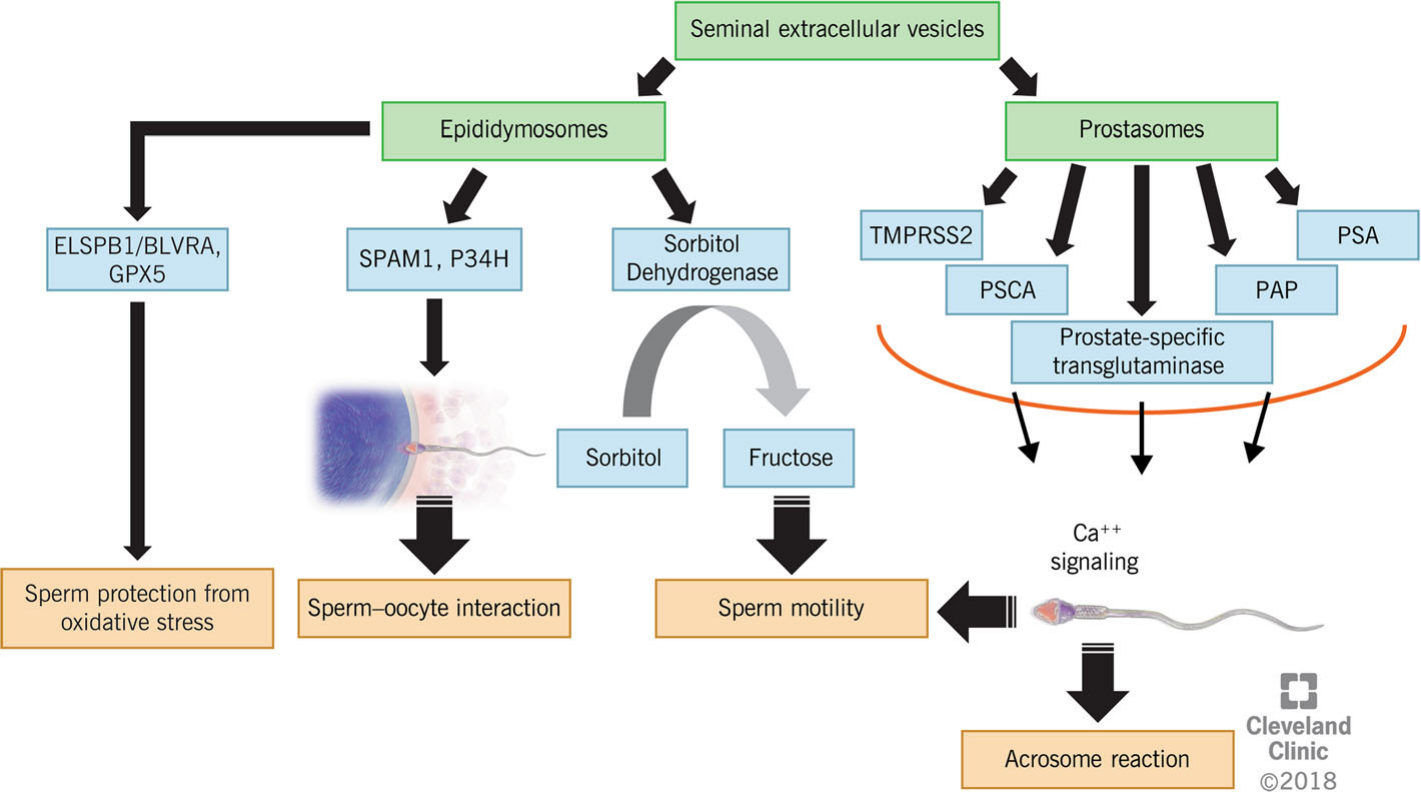
Role of seminal extracellular vesicles, epididymosomes and prostasomes, and their proteome on spermatozoa function

SP contains a heterogeneous population of microvesicles called exosomes. Seminal exosomes include mainly tetraspanin CD9-positive epididymosomes and prostasomes (Fig. 2), which account for 3% of the total protein complement of SP [43]. Transcription and translation are almost silent in mature spermatozoa due to DNA packaging during spermatogenesis. Besides the PTMs that occur during sperm maturation along the male reproductive tract, exosomes play an important role in supporting sperm functions [44]. Exosomes are RNA and protein containing microvesicles, which like viruses, originate from inside the cells and are released into the extracellular compartment. This is in contrast to other microvesicles that are pinched off from the plasma membrane [45]. Genetic material can be delivered into target cells via exosomes including protein-encoding mRNAs and small noncoding RNAs involved in regulation of gene expression [46–48]. A recent comprehensive proteomic analysis by Yang et al. [49] reported a total of 1474 proteins in human seminal exosome samples. Through gene ontology (GO) analysis, the authors showed that seminal exosomes-associated proteins were mostly linked to ‘exosomes,’ ‘cytoplasm,’ and ‘cytosol’, which are involved in biologic processes such as metabolism, energy pathways, protein metabolism, cell growth and maintenance, and transport [49]. SP transmits a plethora of proteins to the spermatozoa via exosomal-binding that are important for maturation and protection of the male gametes, as well as for the maintenance of their function.

Epididymosomes have a complex proteome and their composition changes along the epididymis. Moreover, the epididymosomes present in the ejaculate do not significantly contribute to the population of exosomes present in the ejaculate. These suggests that epididymosomes transfer their contents to the spermatozoa during their transit through the epididymis. Proteins associated to epididymosomes are not processed through the endoplasmic reticulum–Golgi apparatus secretory vesicles, have unusual glycosylation and lack N-terminal signal peptides. Additionally, some of them are strongly glycosylphosphatidylinositol (GPI)-anchored to these membranous vesicles. There are basically two subpopulations of epididymosomes, those enriched with epididymal sperm binding protein 1 (ELSPBP1) and the CD9-positive epididymosomes (Fig. 2). ELSPBP1, first described as HE12 in humans, was associated with dead and dying spermatozoa in the ejaculated semen. This protein in association with biliverdin reductase A (BLVRA) uses NADPH as a proton donor to reduce biliverdin to bilirubin. Bilirubin then uses ROS to regenerate biliverdin in the presence of Zn^2+^. Thus, the binding of ELSPBP1/BLVRA complex to dying spermatozoa acts as a scavenger of ROS generated by dying spermatozoa, thereby protecting the surviving spermatozoa from oxidative damage [50, 51]. CD9-positive epididymosomes preferentially bind to or fuse with live spermatozoa and are involved in epididymal sperm maturation. HE5, which is specifically expressed by the human epididymis, is also a human lymphocyte surface protein (CD52) and is GPI-anchored to the sperm surface during the epididymal transit. It is proposed that this protein is transferred to the sperm surface by epididymosomes and is thought to be associated with human immunological infertility [50, 52]. Other epididymosome-associated proteins that are also transferred to sperm surface during epididymal maturation include: enzymes of the polyol pathway, macrophage migration inhibitory factor (MIF), type 5 glutathione peroxidase (GPX5), the sperm adhesion molecule 1 (SPAM1 or PH-20), and the zona pellucida binding protein P34H. Two enzymes of polyol pathway, aldose reductase and sorbitol dehydrogenase are responsible for the generation of fructose by oxidation of sorbitol to fuel sperm motility. MIF is a cytokine that modulates sperm motility during the transit along the male reproductive tract and GPX5 GPX5 might protect the transiting epididymal spermatozoa against OS. Finally, SPAM1 and P34H play a key role in sperm-egg binding [53].

Prostasomes are well-characterized markers of SP function. Examples of prostasomes in human SP include PAP, prostate specific antigen (PSA), type 2 transmembrane serine protease (TMPRSS2), prostate-specific transglutaminase (pTGase) and prostate stem cell antigen (PSCA) (Fig. 2) [54, 55]. These seminal exosomes transfer Ca^2+^ signaling tools namely, progesterone receptors, cyclic adenosine diphosphoribose (cADPR)-synthesizing enzymes, ryanodine receptors (RyRs), and other related molecules to the sperm neck, thus triggering sperm motility and acrosome reaction [56]. By binding to sperm cells, these proteins are able to control the timing of early and late capacitation events to avoid premature induction of the acrosome reaction. Studies have reported that prostasomes help to increase intracellular cAMP which then stimulates protein kinase A (PKA) to indirectly induce tyrosine phosphorylation to activate capacitation and reorganization of plasma membrane in the spermatozoon [57–61]. In vitro studies have shown that prostasomes can be responsible for transferring cholesterol to spermatozoa during end-stage capacitation [60, 62]. In addition, interactions of prostasomes within the female reproductive tract cause local modulation of the immune system and hence, prevent spermatozoa from being phagocytosed, damaged or killed by the attacks of neutrophil granulocytes [62].

### Protective role of seminal plasma molecules in fertilization

In semen, both leukocytes and immature/abnormal spermatozoa are intracellular sources of ROS. If uncontrolled, ROS can lead to damages in spermatozoa proteins, lipids and DNA. Spermatozoa are particularly susceptible to oxidative damage due to the high content of polyunsaturated fatty acids (PUFAs) in their plasma membrane and also the limited inner antioxidant system [24, 63, 64]. However, SP has an efficient antioxidant defense system that protects spermatozoa during ejaculation and the first steps of their journey through the female reproductive tract [65]. SP has a great repertoire of inflammatory molecules obtained from the testes, including macrophages, somatic cells (Leydig and Sertoli cells), spermatogonia, leukocytes and mesenchymal cells [66, 67]. The secretion of seminal cytokines is also carried out by immunocompetent cells in various pathogenic conditions including leukocytospermia [68]. Cytokines are small proteins that are important in cell signaling and play a key role in the regulation of fertilization. The accumulation of cytokines and their soluble receptors such as interleukins (IL-1, IL-2, IL-4, IL-6, IL-8, IL-10, IL-12, IL-13, IL-17, IL-18), tumor necrosis factor-alpha (TNF-α), tumor necrosis factor receptor 1 and 2 (TNFR1/2), interferon-gamma (IFN-γ) and granulocyte colony-stimulating factor (G-CSF) in the SP has been observed in semen samples of both fertile and infertile men [38, 66, 67, 69]. A study by Seshadri et al. [69] confirms that cytokines seldom act in isolation and may affect sperm function directly or indirectly by acting as members of a network with other cytokines. Their results also suggest that the cytokines may not originate from the testis as their level is observed to be augmented in the obstructed azoospermic group.

SP can modulate immune responses in the male and female reproductive tracts (Table 3). During intercourse, a normal influx of leukocytes is important for the interaction of spermatozoa with the female tract [70]. This early leukocyte attack allows the female reproductive system to eliminate spermatozoa with poor fertilizing capacity based on poor DNA integrity [71]. However, the most capable spermatozoa to fertilize have to escape these harsh uterine conditions very quickly to avoid immune opposition by the female reproductive tract after their detachment from the SP microenvironment [71–73]. TGF-β in human seminal plasma plays a key role in inducing proinflammatory cytokine synthesis and leukocyte recruitment by the cervical cells [74]. Some of the proinflammatory cytokines produced by human cervical and vaginal epithelial cells after interaction with SP include granulocyte-macrophage colony-stimulating factor (GM-CSF), IL-1, IL-6 and IL-8. They also produce some chemokines, such as the monocyte chemoattractant protein-1 (MCP-1) [75, 76]. These molecules contribute to the recruitment of macrophages, dendritic cells and lymphocytes into the epithelial layers and deeper stromal tissues [5, 70, 76].

**Table 3:** Seminal plasma proteins in spermatozoa protection, transport and interaction with the female reproductive tract

Proteins involved	Site of Action	Mechanism of action	Reference
FOXP3	Uterine regulatory T cells	Immunosuppression and improved tolerance towards paternal antigens	[96–98]
GM-CSF	Embryo	Blastocyst stage development	[72]
IL-6	Embryo	Protection from apoptosis by secreting anti-apoptotic micro RNAs	[72]
LIF	Inner cell mass	Blastocyst development	[72]
IGF-1	Germ cells	Maturation of spermatozoa	[27, 28]
α_2_-macroglobulin	Germ cells	Progressive motility	[27, 28]
Enkephalin	Sperm cells	Sperm motility	[27, 28]
VEGF, MMPs	Endometrium	Embryo implantation	[40, 99]
TGF-β, PGE	Female reproductive epithelial tissues	Inflammatory signaling response	[40, 99]
GM-CSF, IL-1A, IL-6, IL-8, MCP-1 (CCL2), MIP3A (CCL20)	Female epithelial layers and deeper stromal tissues	Immediate and rapid influx of inflammation to cause fertilization	[5, 65, 74, 99]

Several studies showing the importance of seminal plasma proteins in the regulation of male and female reproductive mechanisms. However, some of the studies were conducted with animal samples and still need validation by studies with human samples

*Abbreviations*: *FOXP3* forkhead box P3, *GM-CSF* granulocyte-macrophage colony stimulating factor, *IL* interleukin, *LIF* leukemia inhibitory factor, *IGF* insulin like growth gactor, *VEGF* vascular endothelial growth factor, *MMPs* matrix metalloproteinases, *TGF-β* transforming growth factor beta, *PGE* prostaglandin E synthase, *MCP-1* monocyte chemoattractant protein-1, *MIP-3A* macrophage inflammatory protein 3, *CCL* chemokine ligand

Additional studies have shown that the role of SP is not only restricted to the cervix, but it also extends to the uterus [76, 77]. Uterine receptivity is entirely dependent on a healthy uterine environment to encourage embryonic implantation [78]. Seminal constituents bound to the post-acrosomal region of the sperm head are carried in conjunction with the sperm to the higher parts of the female tract. Along with the peristaltic contractions transporting macromolecular materials, unique vascular connections facilitate the transport of progesterone and inflammatory mediators from the cervix to the endometrial tissue [40]. IL-1 and TNF-α have been found to stimulate progesterone and prostaglandin synthesis to regulate gonadotropin secretion for corpus luteum function [79]. Seminal fluid helps in the regulation of endometrial tissue remodeling and uterine receptivity by recruiting leukocytes, especially macrophages. These are further mediated by adhesion ligands such as integrins, selectins, cadherins and immunoglobulins, thereby losing the inhibitory components that may act as a barrier to an attaching embryo [40, 76, 80]. The expression of epididymal protein, E-cadherin in the SP was found to be responsible for sperm-oocyte adhesion [81]. Clinical studies on SP insemination in couples undergoing in vitro fertilization (IVF) or intracytoplasmic sperm injection (ICSI) have shown a significant improvement in embryo transfer after exposure to SP [82, 83]. Human studies indicated that when embryos were cultured in the presence of GM-CSF, blastocyst development rate was increased, leading to increased cell numbers both in the inner cell mass and trophectoderm [84]. Thus, SP plays a key role in spermatozoa protection from oxidative damage, spermatozoa function and interaction with the female reproductive tract. However, further studies are needed to clarify the role of SP in fertilization and contribution to a successful pregnancy using ART.

### Seminal plasma as a source of biomarkers and targeted drug delivery

SP is a complex body fluid, containing a large diversity of proteins. The exact number of proteins expressed by the SP is not known, but possibly up to 10,000. SP proteomics is regarded as a great resource for the discovery of potential biomarkers to improve diagnosis or classification of a wide range of male reproductive disorders. However, the major limitations are the complexity of SP proteome, the inter- and intra-individual variation and the probability of the low abundant proteins to be masked in the presence of very high abundant proteins like semenogelins. This implies that the discovery of low abundance proteins with a critical role in reproductive functions could be facilitated by a selective removal of high abundant proteins using specific methods such as immunoprecipitation. For instance, the removal of the top high abundant proteins from blood by a single-step purification method using a mixer of high-specificity polyclonal antibodies (MARS) is commercially available [85]. Additionally, extracellular vesicles such as exosomes were isolated from small volumes of human blood plasma by Protein Organic Solvent Precipitation (PROSPR) [86]. Similar methods should be applied to SP samples to clarify the role of low abundance SP proteins in male fertility.

Seminal exosomal vesicles may be developed for targeted delivery of drugs, vaccines and anti-virals. Recent in vivo studies indicated specific cell tropism by exosomes, which based on their unique characteristics and origin, can be targeted to tissues and/or organs [87–90]. Intravenous injection of exosomes expressing a neuron-targeting protein on their surface into mice was reported to elicit specific gene knockdown in the brain [91]. Furthermore, in murine model of Parkinson’s disease, administration of autologous macrophage-derived exosomes loaded with catalase, produced potent anti-inflammatory and neuroprotective effects [92]. Modification of exosomal membrane is feasible and can be developed to enhance their targeting capability and specificity [93]. It was possible to target exosomes expressing an epidermal growth factor receptor (EGFR)-binding peptide to tumor cells [94]. The major envelope of Epstein–Barr-Virus protein gp350 incorporated into the exosomal membrane target B cells and resulted in improved interaction between helper T cells and B cells [95]. Exosome-mediated therapy may ultimately lead to in vitro maturation of gametes and improvement of ART outcomes. However, more studies need to be conducted to support these early-stage discoveries and develop an interesting technique from the clinical point of view.

## Conclusion

Diagnostic tools to assess male infertility are limited, particularly for investigation of idiopathic infertility. Therefore routine semen analysis is still the basis for evaluation of a subject’s fertility status. However, as semen analysis offers a rough assessment of male fertility, additional diagnostic tools are needed in a clinical setting. Emerging ‘omics studies’ in general and high throughput proteomics of SP in particular, holds promise for the development of novel biomarkers. These biomarkers could be useful in the evaluation of natural fertility, differentiating between the various infertility etiologies, and predicting ART success. Nevertheless, a closer look into the seminal exosomes, natural nanocarriers, could bring new insights for targeted drug delivery for new therapies.

## Abbreviations

2D-DIGE: Two-dimensional difference gel electrophoresis
ART: Assisted reproductive technologies
B2M: Beta-2-microglobulin
cADPR: Cyclic adenosine diphosphoribose
CNDP2: Cytosolic nonspecific dipeptidase
ECM1: Extracellular matrix protein 1
FAM3D: Family with sequence similarity 3 member D
G-CSF: Granulocyte colony-stimulating factor
GM-CSF: Granulocyte-macrophage colony-stimulating factor
GO: Gene ontology
GPI: Glycosylphosphatidylinositol
GPX5: Type 5 glutathione peroxidase
hCAP18: Human cationic antimicrobial protein
ICSI: Intracytoplasmic sperm injection
IFN-γ: Interferon-gamma
IL: Interleukin
IVF: In vitro fertilization
LC: Liquid chromatography
LDHC: Lactate dehydrogenase C
LGALS3BP: Galectin 3 binding protein
MALDI: Matrix-assisted laser desorption/ionization
MCP-1: Monocyte chemoattractant protein-1
MIF: Macrophage migration inhibitory factor
MIP: Macrophage inflammatory protein
MME: Membrane metalloendopeptidase
MS: Mass spectrometry
MS/MS: Tandem mass spectrometry
PAP: Prostatic acid phosphatase
PGDS: Prostaglandin-D synthase
PGK2: Phosphoglycerate kinase 2
PIP: Prolactin-inducible protein
PKA: Protein kinase A
PSA: Prostate specific antigen
PSCA: Prostate stem cell antigen
PSMA3: Proteasome subunit alpha type-3
pTGase: Prostate-specific transglutaminase
PTMs: Post-translational modifications
PUFAs: Polyunsaturated fatty acids
ROS: Reactive oxygen species
RyRs: Ryanodine receptors
SP: Seminal plasma
SPAM1: Sperm adhesion molecule 1
TEX101: Testis-expressed protein 101
TGF-β: Transforming growth factor beta
TKTL1: Transketolase-like protein 1
TMPRSS2: Type 2 transmembrane serine protease
TNFR1/2: Tumor necrosis factor receptor 1 or 2
TNF-α: Tumor necrosis factor-alpha
TOF: Time-of-flight
